# Education and Atrial Fibrillation: Mendelian Randomization Study

**DOI:** 10.5334/gh.1107

**Published:** 2022-03-29

**Authors:** Yaozhong Liu, Chan Liu, Qiming Liu

**Affiliations:** 1 Department of Cardiovascular Medicine, Second Xiangya Hospital, Central South University, Hunan Province, China; 2 International Medical Department, Second Xiangya Hospital, Central South University, Hunan Province, China

**Keywords:** Atrial fibrillation, Education, Mendelian randomization

## Abstract

Low social-economic status is associated with atrial fibrillation (AF), but the extent of any causative effect is unclear. In the present study, we evaluated the causal role of educational attainment (EA) on AF using Mendelian randomization (MR) analysis. Results from traditional single-variable MR indicated a modest causal effect of EA on AF. Sensitivity analyses using different MR methods yielded consistent results. Multi-variable MR and mediation analysis revealed that the protective effect of higher EA on AF was partially mediated by reducing cardiometabolic risk factors and smoking behavior. Our findings suggest that extending education, for example increasing school-leaving age, could lower the global burden of AF.

Atrial fibrillation (AF) is the most common arrhythmia which poses a significant burden to clinicians and patients. Whether higher educational attainment (EA) decreases the risk of AF remains unclear. Mendelian randomization (MR) is a powerful strategy for evaluating causality, which exploits the principle that genotypes should be immune to confounding and reverse causation bias, and has been used to infer the causal effects of EA on coronary artery disease and stroke [1]. We applied the MR approach to assess the causal relationship between EA and AF. We obtained AF genetic association summary statistics from the largest genome-wide association study (GWAS) [2], which included 60,620 cases and 970,216 controls of European ancestry.

The GWAS for EA [3], measured as the number of years of schooling completed, included 1,131,881 individuals of European ancestry. We selected single-nucleotide polymorphisms (SNPs) associated with EA at genome-wide significance (P < 5 × 10^–8^) in the meta-analysis of 766,345 individuals (standard deviation [SD] = 4.6 years), which excluded participants from 23andMe (the summary data containing 23andMe participants is not made publicly available according to privacy protection agreement). These SNPs were clumped with a 10,000 kB window to a threshold of linkage disequilibrium (LD) R2 < 0.001 to ascertain independence. If an SNP was unavailable in the outcome data set, the proxy SNP (R2 > 0.8) was identified. Finally, 317 SNPs were included in the main analysis.

In the conventional MR analysis (inverse-variance weighted method), 1 SD longer schooling years was associated with a 14% lower risk of AF (odds ratio [OR], 0.86; 95% confidence interval [CI], 0.79–0.94; P = 6.6 × 10^–4^). The Egger-intercept test indicated no directional pleiotropy (P = 0.91). As predicted, sensitivity analyses using weighted median MR and MR-Egger provided less precise estimates. However, their causal estimates were virtually identical in terms of direction and magnitude (***Figure 1***). After correcting for horizontal pleiotropic outliers, the MR Pleiotropy Residual Sum and Outlier (PRESSO) method yielded similar results. We also conducted a reverse MR analysis and found no significant association between genetically determined AF and EA.

**Figure 1:**
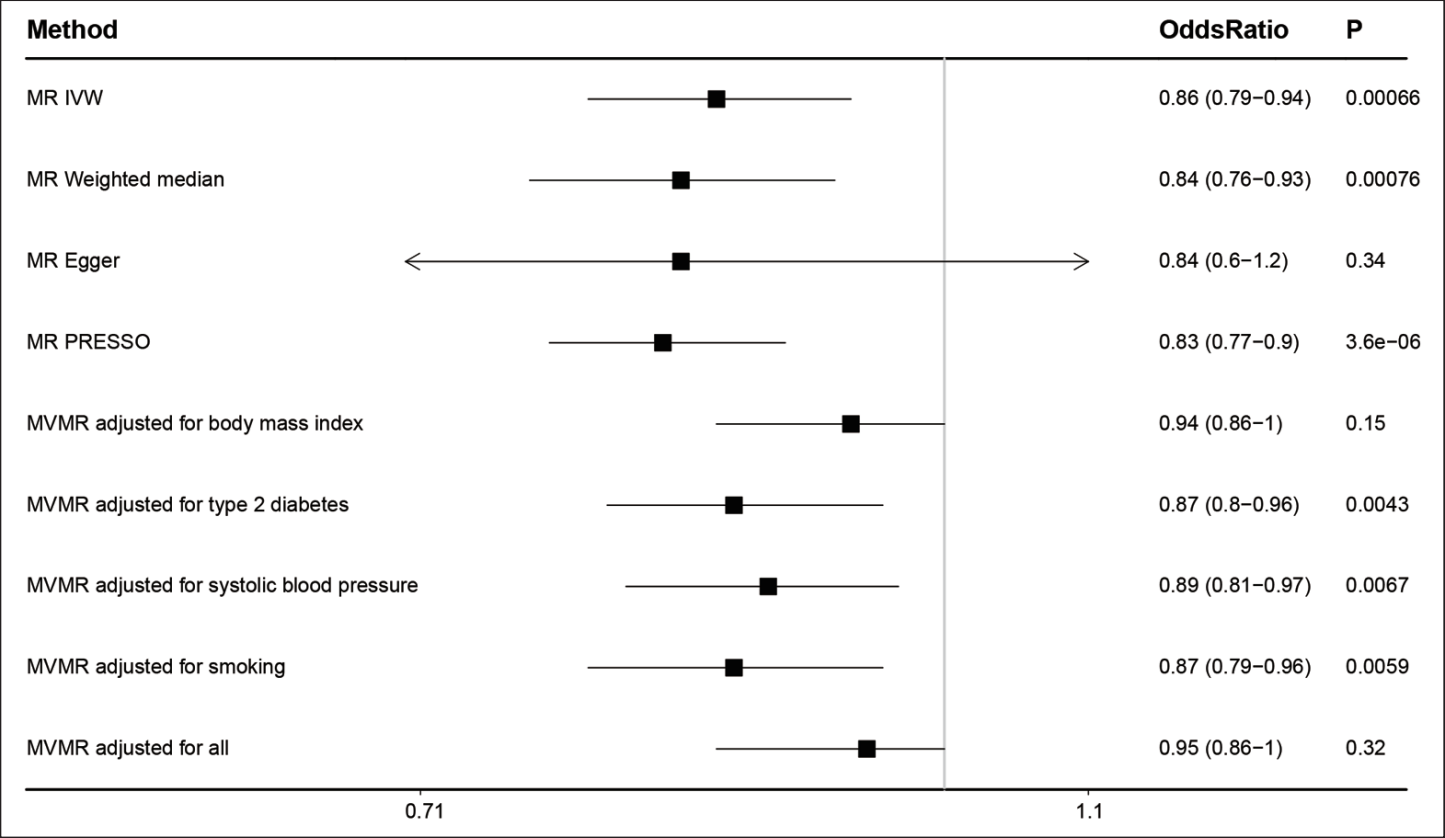
**Association between genetically determined educational attainment and atrial fibrillation.** Odds ratios are expressed per 1-SD (4.6 years) longer schooling years. MR, mendelian randomization; IVW, inverse variance–weighted; PRESSO, Pleiotropy Residual Sum and Outlier; MVMR, multivariable MR.

As the UK Biobank (UKB) individuals participated in both the EA and AF GWASs, which could cause weak instrumental bias due to sample overlap, we conducted secondary analyses. Firstly, the genetic associations of the 317 SNPs with EA were retrieved from an earlier EA GWAS (n = 328,917, SD = 4.2 years) that had a smaller portion of UKB participants (n = 111,349) [4]. This analysis yielded similar results (OR, 0.86; 95%CI, 0.79–0.95; P = 0.002). Secondly, we used the latest FinnGen 2021 AF GWAS (22,068 cases and 116,926 controls) as the outcome data, this analysis also estimated a negative effect of EA on AF which had a larger magnitude (OR, 0.67; 95%CI, 0.57–0.78; P = 4.5 × 10^–7^).

The association between low EA and AF risk may be mediated by increased cardiometabolic risk factors and unhealthy behaviors [5]. Previous MR analyses have shown that genetic liability to EA is negatively associated with body mass index (BMI), type 2 diabetes (T2D), systolic blood pressure (SBP), and smoking behavior [6]. Using the multivariable MR, we conducted a mediation analysis. The proportion of the total effect of EA on AF mediated through BMI, T2D, SBP, and smoking was 57.5%, 9.8%, 18.7%, and 7.1%, respectively; and all four risk factors combined were estimated to mediate 66.8% of the total effect. In MVMR adjusted for BMI and for all four, the relationship between EA and AF was no longer significant. These results suggest that intervening on these traditional cardiovascular risk factors would reduce cases of AF attributable to lower levels of education. Nevertheless, more than 30% of the protective effect of education remains unexplained. Further research identifying other related factors, such as negative emotions that might cause alterations in autonomic nervous system, will be key to reducing social inequalities in AF.

The strength of this study is the MR design and the use of the largest GWASs of exposure and outcome. Another strength is that individuals from GWASs included in this analysis are of European descent, which diminished bias caused by population stratification.

This study has limitations. The results from an MR study can be violated by pleiotropy, which describes a genetic variant that is associated with multiple traits. However, the overall causal estimate based on all genetic variants is unlikely to be biased, as limited evidence supports the existence of directional pleiotropy, and sensitivity analyses yielded consistent results. Another shortcoming is that potential dynastic effects may also violate the MR assumptions. Future research is recommended to conduct a within-family analysis for reducing such bias [7]. Finally, GWAS and MR studies conducted in other populations, such as Asians, are expected to generalize our conclusions.

In summary, genetic evidence based on MR approaches suggests a causal effect of EA on AF, the effect of which was partially mediated by cardiometabolic risk factors and smoking behavior. The finding suggests that extending education, for example by increasing school-leaving age, could make the population at a lower risk of AF. Public health initiatives (eg, screening of AF, management of risk factors) should be developed with a specific focus on the low-educated vulnerable group to reduce burden of this increasingly common heart disease.
